# Ripening cycle and its relationship with fruiting in advanced *Coffea arabica* L. progenies derived from intra- and interspecific crosses

**DOI:** 10.3389/fpls.2026.1860074

**Published:** 2026-07-14

**Authors:** Carlos Andres Unigarro, Aquiles Enrique Darghan, Daniel Gerardo Cayón Salinas, Claudia Patricia Flórez-Ramos

**Affiliations:** 1 Discipline of Plant Physiology, National Coffee Research Center, Cenicafé, Manizales, Colombia; 2 Departamento de Agronomía, Facultad de Ciencias Agrarias, Universidad Nacional de Colombia, Bogotá, Colombia; 3 Departamento de Ciencias Agrícolas, Facultad de Ciencias Agrarias, Universidad Nacional de Colombia, Palmira, Colombia; 4 Plant Breeding, National Coffee Research Center, Cenicafé, Manizales, Colombia

**Keywords:** dynamic fruit load, fruit mass, fruit ripening, growing degree-days, hybridization, leaf area-to-fruit ratio

## Abstract

**Introduction:**

The fruit ripening cycle is a key factor in coffee production because it directly influences harvest scheduling and crop environmental adaptability. Therefore, evaluating the variability of this trait in different progenies and its association with the source–sink relationship and fruit characteristics is fundamental for optimizing production and breeding.

**Methods:**

In coffee progenies derived from intraspecific and interspecific hybridization, the ripening cycle was recorded using growing degree days (GDD) during the main and secondary harvest periods. Concurrently, variables related to climate, source–sink relationships, fruit characteristics, and fruit load dynamics were recorded. Multivariate clustering and variable selection methods, semiparametric multivariate analysis with repeated measures, and non-parametric longitudinal data analysis were implemented for data interpretation.

**Results:**

The progenies were classified into three clusters. The early- and late-ripening clusters presented the lowest and highest GDD values, respectively, across harvests. In contrast, stable-ripening progenies presented low GDD variation across harvests. The effect of ripening directly impacts the dynamics of the fruit load harvested over time. Variations in the source–sink relationship variables and physical fruit characteristics were attributed primarily to the fruit load. The hybridization origin was not associated with the variability of the ripening cycles.

**Discussion:**

The consistent performance of early- and late-ripening progenies at each harvest, despite variable ripening cycle durations, and the low sensitivity to inter-harvest changes in stable progenies indicate that the progenies respond differently to environmental conditions. This response directly influences the fruit load dynamics. The ripening cycle duration may be indirectly influenced by the source–sink relationship, but not by physical fruit characteristics or hybridization. The latter, due to the sample size, should be interpreted with caution and verified in future studies.

## Introduction

The genus *Coffea* comprises approximately 130 species (Davis and Rakotonasolo, 2021), but only two have global economic relevance: *Coffea arabica* L. (tetraploid, autogamous, adapted to altitudes above 1,000 m, optimal temperature range of 18 °C–21 °C, optimal rainfall range of 1,500–2,000 mm) and *Coffea canephora* Pierre ex Froehner (diploid, allogamous, adapted to altitudes below 1,000 m, optimal temperature range of 22 °C–30 °C, optimal rainfall range of 2,000–3,000 mm) (Akaffou et al., 2014; Campuzano-Duque et al., 2021). These two species account for 56.3% and 43.7% of global coffee production (ICO, 2021). In Colombia, coffee breeding has focused on developing composite varieties, a strategy that aims to leverage genetic diversity within populations to increase the durability of disease resistance under field conditions. Commercial varieties developed by the National Coffee Research Center (Cenicafé) originated primarily from a cross between Caturra and Timor Hybrid (HdT) CIFC 1343 (Van Der Vossen et al., 2015; Flórez et al., 2017).

This germplasm constitutes the genetic basis for several improved varieties with resistance to coffee leaf rust caused by the fungus *Hemileia vastatrix* Berk. & Broome (Basidiomycota, Pucciniales) (Gaitán-Bustamante et al., 2022). As a result of this program, Cenicafé has released 14 resistant varieties, notably Colombia (Castillo and Moreno, 1988), Castillo® (Alvarado et al., 2008; Flórez et al., 2018), and Cenicafé 1 (Flórez et al., 2016). Currently, approximately 88% of the Colombian coffee-growing area is planted with rust-resistant coffee varieties (Gaitán-Bustamante et al., 2022). However, the resistance conferred by HdT may lose its effectiveness over time because of pathogen evolution (Del Grossi et al., 2013; Cortina-Guerrero and Castro-Caicedo, 2015). In systems where genotypes with similar resistance predominate, hosts exert strong selection pressure on *H. vastatrix* populations, favoring the emergence of new races with broader virulence profiles (Guerra-Guimarães et al., 2023).

To maintain the durability of field resistance, the Cenicafé breeding program has implemented strategies to diversify the sources of resistance incorporated into commercial varieties. Since the 1970s, an interspecific hybridization program between *C. arabica* and *C. canephora* has been developed, generating Arabica progenies that incorporate resistance genes from *C. canephora*. This strategy has broadened the genetic basis of resistance and strengthened its longevity under cultivation conditions (Castro Caicedo et al., 2013; Cortina-Guerrero and Castro-Caicedo, 2015; Cortina-Guerrero, 2019). As a result of this breeding process, the Castillo® 2.0 variety was released in 2024 (Flórez et al., 2024). *C. canephora* is a valuable genetic source for breeding programs, as it contributes not only to rust resistance but also to improved agronomic traits, such as yield and hardiness, in *C. arabica* (Ferrão et al., 2019). Recently, the need to develop cultivars that mitigate losses from drought and heat has driven the search for morphological and physiological traits in germplasm banks of breeding programs (DaMatta et al., 2018; Torres et al., 2024).

The introgression of genes for resistance, yield, and hardiness from *C. canephora* to *C. arabica* through a selection process produces progenies that incorporate these traits. However, during this process, the selected genotypes may have unintended characteristics, such as potential changes in the “ripening cycle”. This term refers to the time elapsed between anthesis and fruit ripening, which is typically measured in days after flowering (DAF) (Partelli et al., 2014). This is because the fruit ripening cycle of *C. arabica* (266 DAF) generally requires less chronological time than that of *C. canephora* (284 DAF) (Salazar et al., 2019). In this sense, the classification of coffee cultivars on the basis of the duration of the ripening cycle is crucial for harvest scheduling, quality, and marketing (Bardin-Camparotto et al., 2012; Da Silva Angelo et al., 2019). This is due to the variability in the effects of coffee genotypes and environmental conditions on the fruit ripening cycle (Camargo and Camargo, 2001; Pezzopane et al., 2003; Morais et al., 2008; Petek et al., 2009). In *C. arabica*, the period from anthesis to fruit ripening is, on average, between 220 and 243 DAF (Cannell, 1985; Salazar et al., 1994; Petek et al., 2009; DaMatta et al., 2010; Ocampo et al., 2010; Ferreira et al., 2019), although it can fluctuate between 204 and 266 DAF for a given cultivar under different environmental conditions and altitudinal gradients (Arcila et al., 2007). The ripening cycle also varies between cultivars, with apparent differences between early-ripening (213–227 DAF) and late-ripening (238–254 DAF) cultivars (Da Silva Angelo et al., 2019). These differences are, in many cases, greater than 30 days (Petek et al., 2009; Pezzopane et al., 2012; De Oliveira Aparecido et al., 2018). Conversely, the ripening cycle in *C. canephora* ranges from 216 DAF in early-ripening clones to 300 DAF in late-ripening clones (Partelli et al., 2014; Crasque et al., 2024, 2025).

The ripening cycle can also be described by the relationship between a plant’s development rate and air temperature using growing degree days (GDD) (Bonhomme, 2000), a standardized indicator for monitoring and predicting vegetative development on the basis of the heat accumulation required to complete a specific phenological stage (Paredes et al., 2025). A key factor for calculating the GDD is the threshold or base temperature, which corresponds to the temperature limit below which the growth rate tends to zero (Bonhomme, 2000; Paredes et al., 2025). For *C. arabica*, the Caturra variety required 2,560 GDD to complete the ripening cycle during the secondary harvest and 2,445 GDD during the main harvest (Jaramillo and Guzman, 1984), whereas the Colombia variety (composite) reached ripening at 2,836 GDD for the main harvest (Salazar et al., 1994); in both studies, the base temperature used was 10 °C. In other studies with a base temperature of 10.0 °C, early-ripening cultivars completed the ripening cycle between 2,587 and 2,707 GDD, whereas late-ripening cultivars completed the cycle between 2,845 and 2,935 GDD (Petek et al., 2009). At a base temperature of 10.5 °C, the ripening cycle of late cultivars reached 3,090 GDD (Pezzopane et al., 2012). The ripening cycle of *C. canephora* requires approximately 3,500–3,600 GDD using a base temperature of 10 °C (Salazar et al., 2019). In addition to temperature, rainfall and solar radiation have been associated with fruit ripening cycles. In a study of early-ripening *C. arabica* cultivars, the total precipitation was greater than that for other cultivars, and they required more daily solar radiation during the ripening cycle than late-ripening cultivars, which developed under lower levels of solar radiation over more extended periods with reduced water availability (Da Silva Angelo et al., 2019).

Fruiting in plants depends mainly on the source–sink relationship, physical fruit characteristics, and dynamics of the fruit load over time. In *C. canephora*, early-ripening clones exhibit a high net assimilation rate in the leaves, implying an increase in source strength (Morais et al., 2012). An increase in the net assimilation rate has been associated with a reduction in the leaf area-to-fruit ratio, which occurs with high fruit loads for *C. arabica* (Vaast et al., 2005; DaMatta et al., 2008; Unigarro et al., 2021). Moreover, approximately 20 cm^2^ of leaf area of *C. arabica* is needed to supply each fruit without severely reducing vegetative growth (Cannell, 1974). Under favorable conditions, 12 to 20 fruits per node can grow, with two leaves of 30 to 40 cm^2^ each (Cannell, 1985). Furthermore, reports indicate that for *C. arabica*, plants with low fruit loads are characterized by accelerated fruit ripening, regardless of light conditions (shade or full sunlight) (Vaast et al., 2006). Vegetative growth rates for branches, height, and the stem (diameter) decrease when the leaf area-to-fruit ratio decreases (Unigarro et al., 2021; León-Burgos et al., 2024). Specific leaf area is inversely associated with the leaf area-to-fruit ratio and with fruit load (Bote and Jan, 2016; León-Burgos et al., 2024). Some physical indicators, such as fruit fresh mass, fruit size, and bean dry mass, decrease when the leaf area-to-fruit ratio decreases (high fruit loads) for *C. arabica* (DaMatta et al., 2008; Bote and Jan, 2016; León-Burgos et al., 2024).

In Colombia, latitude influences the magnitude of flowering events during the flowering period (FP) (FP-I, November–April and FP-II, May–October); notably, above 4° N latitude, the most abundant flowering occurs during FP-I, whereas below this latitude, it occurs during FP-II (Unigarro et al., 2023). The sum of flowering events in each FP determines the fruit load per harvest, resulting in a main harvest with a high fruit load and a secondary harvest with a low fruit load. Furthermore, the distribution of flowering within each stage influences the fruit load dynamics during each period. In the central coffee-growing region (4–5° N latitude) of Colombia, fruits resulting from flowering events in FP-I constitute the main harvest (between July and December), representing 60%–70% of the total fruits harvested per year, whereas the secondary harvest (between January and June) represents 30%–40% of the annual total, corresponding to flowering events in FP-II (Trojer, 1954; Arcila et al., 2007; Rendón, 2020). These changes in fruiting for each harvest may modify the source–sink relationship and physical characteristics of the fruit, factors that have not yet been investigated in detail.

In this study, changes in the ripening cycle duration of coffee fruits associated with the advanced and improved progenies of the Cenicafé Breeding Program and their association with factors such as the source–sink relationship, physical fruit characteristics, hybridization origin, and fruit load dynamics during harvest are investigated. On the basis of these findings, the following hypotheses are proposed: i) the duration of the fruit ripening cycle for the evaluated progenies is sufficiently variable to form ripening clusters; ii) fruiting, assessed via the source–sink relationship and physical characteristics of the fruit, is linked to ripening clusters; iii) changes in fruiting between harvests, represented by the source–sink relationship, are linked to the duration of the fruit ripening cycle; iv) fruiting dynamics during harvest are linked to ripening clusters; and v) hybridization type is linked to the duration of the fruit ripening cycle. This information is useful for understanding the relationship between the duration of the fruit ripening cycle and plant growth dynamics, which is necessary for developing composite varieties with synergistic ripening.

## Materials and methods

### Location and plant material

The study was conducted at the Naranjal Experimental Station (Chinchiná, Caldas, Colombia; 4°58′19.1″N, 75°39′8.2″W; 1,407 m a.s.l.) located in the central coffee-growing region of Colombia (FNC, 2017). The Breeding Program of the National Coffee Research Center (Cenicafé) established 36 advanced coffee progenies in experimental plots consisting of seven plants (five effective plants and two as borders) using a block design, with three blocks associated with each slope, in November 2020 at a planting density of 7,142 plants/ha (1.4 m between rows and 1.0 m between plants in a rectangular arrangement). The evaluated progenies corresponded to intraspecific hybridization crosses of *C. arabica* (Intra.H.) and interspecific hybridization crosses between *C. arabica* and *C. canephora* (Inter.H.) with a high degree of Arabization, as indicated by the filial generation (**Table 1**). During the phenological cycle, soil fertilization was performed according to the nutritional requirements of the crops (Sadeghian, 2014), and integrated weed, pest, and disease management was performed according to the technical recommendations established by Cenicafé (2021). In each plot, two effective plants were randomly selected for evaluation during the secondary harvest (SH) and main harvest (MH) periods. The phenological stages of the coffee plants were identified using the extended Biologische Bundesanstalt, Bundessortenamt und CHemische Industrie (BBCH) scale (Arcila et al., 2002).

**Table 1:** Progeny and treatment number of progenies derived from intraspecific *Coffea arabica* crosses (Intra.H.) and interspecific crosses between *C. arabica* and *Coffea canephora* (Inter.H.).

Hybridization | progeny	#	Crossing	F
Intra.H.			
MEG102014(2017-2) #295	1	Etiopía × (Caturra × Timor hybrid)	F5
MEG102004(2010-6) #47	7	Timor hybrid × (Caturra × Timor hybrid)	F5
MEG105001(LIBANO 7x7) #1359	38	Caturra × Timor hybrid	F8
MEG105001(LIBANO 7x7) #1472	29	Caturra × Timor hybrid	F7
MEG102014(2017-2) #1949	6	Caturra × Timor hybrid	F7
MEG105001(2013-2) #48	30	Caturra × Timor hybrid	F8
MEG105001(2013-2) #706	31	Caturra × Timor hybrid	F8
Inter.H.			
MEG105001(LIBANO 8x8) #304	20	[Caturra × (Caturra × *C. canephora*)] × [Catuaí × (Caturra × Borbón)]	F5
MEG105001(LIBANO 8x8) #326	25	[(Caturra × Timor hybrid) × (Caturra × Timor hybrid)] × [Catuaí × (Caturra × Borbón)]	F5
MEG105001(LIBANO 8x8) #380	22	[Caturra × (Caturra × *C. canephora*)] × [Catuaí × (Caturra × Borbón)]	F5
MEG105001(LIBANO 8x8) #407	21	[(Caturra × Timor hybrid) × (Caturra × Timor hybrid)] × (Sudán Rume × Catuaí)	F5
MEG105001(LIBANO 8x8) #571	27	[(Caturra × Timor hybrid) × (Caturra × Timor hybrid)] × Etiopía	F5
MEG105001 BLONAY #170,173	63	(Caturra × *C. canephora*) × Caturra	F6
MEG105001(LIBANO 8x8) #123	42	Caturra × [(Caturra × *C. canephora*) × Caturra]	F5
MEG105001(LIBANO 8x8) #139	43	(Caturra × *C. canephora*) × Caturra	F7
MEG105001(LIBANO 8x8) #290	51	(Caturra × *C. canephora*) × Caturra	F7
MEG105001(LIBANO 8x8) #469	52	(Caturra × *C. canephora*) × Caturra	F7
MEG105001(LIBANO 8x8) #601	41	Caturra × [(Caturra × *C. canephora*) × Caturra]	F5
MEG105001(LIBANO 8x8) #615	44	(Caturra × *C. canephora*) × Caturra	F7
MEG102003(2009-17) #109	86	(Caturra × *C. canephora*) × Caturra	F5
MEG102003(2009-17) #13	95	(Caturra × *C. canephora*) × Caturra	F5
MEG105001(2013-3) #1511	75	(Caturra × *C. canephora*) × Caturra	F6
MEG105001(2013-2) #165	64	(Caturra × *C. canephora*) × Caturra	F6
MEG102003(2009-17) #250	80	(Caturra × *C. canephora*) × Caturra	F5
MEG102003(2009-17) #300	84	(Caturra × *C. canephora*) × Caturra	F5
MEG105001(2013-2) #552	70	(Caturra × *C. canephora*) × Caturra	F6
MEG102003(2009-17) #561	102	(Caturra × *C. canephora*) × Caturra	F5
MEG102003(2009-17) #572	82	(Caturra × *C. canephora*) × Caturra	F5
MEG102003(2009-17) #583	99	(Caturra × *C. canephora*) × Caturra	F5
MEG102003(2009-17) #601	90	(Caturra × *C. canephora*) × Caturra	F5
MEG105001(2013-2) #698	58	(Caturra × *C. canephora*) × Caturra	F6
MEG105001(2013-2) # 718	68	(Caturra × *C. canephora*) × Caturra	F6
MEG105001(2013-2) #84	66	(Caturra × *C. canephora*) × Caturra	F6
MEG102003(2009-17) #86	93	(Caturra × *C. canephora*) × Caturra	F5
MEG105001(2013-2) #98	73	(Caturra × *C. canephora*) × Caturra	F6
MEG105001(2013-2) #305,493	54	(Caturra × *C. canephora*) × Caturra	F6

F, filial generation.

### Fruit ripening cycle and climatic variables

Two plagiotropic branches from the middle canopy were selected for each effective plant per progeny. On these branches, five nodes that exhibited greater than 70% anthesis (BBCH stage 60) during the peak flowering event in both the SH (September 6, 2023) and the MH (February 12, 2024) were marked, and the event date was recorded. The flowering peak was defined by weekly records made at the experimental station according to the protocol described by Rendón et al. (2008). Monitoring of fruit development from anthesis (BBCH stage 60) consisted of monthly visual inspections for potential mechanical and biological damage for up to 200 DAF. At this point, the observation frequency increased to twice per week to record the date when more than 80% of the fruits per node from the peak flowering event were ripe (BBCH stage 88), as identified by the characteristic cherry-red color of the fruit epidermis. Fruit ripeness recording began on April 22, 2024, and ended on May 28, 2024, for the SH; for the MH, it started on September 12, 2024, and concluded on October 30, 2024. The chronological time for fruit ripening (CTFR) corresponds to the number of days elapsed from the flower anthesis date (BBCH stage 60) to the date the fruit reached a ripe state (BBCH stage 88), with DAF used as the unit of measurement.

The fruit ripening cycle was evaluated using the GDD recorded during the CTFR for each progeny and harvest according to Equation 1. This assessment accounts for the influence of environmental temperature on the phenological expression of the CTFR for a given genotype (Arcila et al., 2007; Flórez et al., 2013; Paredes et al., 2025). Additionally, photosynthetically active radiation (PAR), rainfall (R), relative humidity (RH), and average temperature (T) were recorded daily during the CTFR for each progeny and harvest period. PAR and R were calculated as the sum of daily values, whereas RH and Temp were obtained as average values. Daily meteorological conditions were monitored in the field using a RAWS-F remote automatic weather station (FireWeather, Campbell Scientific^®^, Logan, Utah, USA).

(1) $$GDD=\;\;\overset{n}{\underset{i=1}{\sum }}\frac{Tmax+Tmin}{2}-Tb$$

where GDD represents the accumulated number of growing degree days (on a daily basis); Tmax represents the daily maximum air temperature (°C); Tmin is the daily minimum air temperature (°C); Tb is the base temperature (°C), with a value of 10.0 °C for coffee (Rodríguez et al., 2011); and n is the number of days between the date of anthesis and the date of fruit ripening. Additionally, GDD was calculated using a Tb of 10.5 °C (Pezzopane et al., 2008) (**Supplementary Table 1**). Base temperatures of 10.0 °C and 10.5 °C were selected for physiological studies conducted in Colombia and Brazil, respectively, to facilitate comparisons. No lower base temperature values were recorded in the daily air temperature values.

### Source–sink relationship during fruit ripening

One week after anthesis (BBCH stage 60) [ $t_{i}$] and 1 week after fruit ripening (BBCH stage 88) [ $t_{f}$], the lengths of the previously selected plagiotropic branches were measured for each effective plant for both the SH and MH. Data recorded at both measurement points for each harvest were used to calculate the variable absolute growth rate of branch length (GBL). The absolute growth rate was calculated using Equation 2 (Hunt, 1990).

(2) $$G_{\left[i,f\right]}=\frac{\left(V_{f}-V_{i}\right)}{\left(t_{f}-t_{i}\right)}$$

where $G_{\left[i,f\right]}$ is the absolute growth rate within the measurement interval and $V_{.}$ is the value of the response of interest at the boundaries of the time interval of $t_{i}$ and $t_{f}$.

Once the fruit ripening date (BBCH stage 88) was recorded for each effective plant, the leaves on the selected plagiotropic branches were measured at each harvest. For each leaf, the length (L) was measured from the apex to the point where the blade meets the petiole, and the width (W) was measured from the right to the left edge of the leaf lamina at its widest point using a ruler. These measurements were used to estimate leaf size (L_S_) using the “Montgomery” model in its probabilistic form (L_S_ = α LW; a deterministic model, although the observation is stochastic). The α parameter used for each progeny was estimated in a previous study (Unigarro et al., 2025b). The number of fruits on each branch was counted, regardless of their ripening stage. The leaf area-to-fruit ratio per branch (LFR) was calculated as the sum of all individual L_S_ values along the branch divided by the total number of fruits on the same branch. The number of fruits per node (F_Node) was determined as the median of the fruit counts of the marked nodes of the selected branches. After the leaf dimensions were measured and the fruits were counted, three leaves per plagiotropic branch were collected. In the laboratory, five 1-cm^2^ circles were extracted from each leaf. The circles were heated to 50 °C in an oven for 72 h until they reached a constant dry mass. The specific leaf area (SLA) was calculated as the ratio of the leaf surface area (circles) to dry mass (Hunt, 1990). The fruit load in the plot was recorded during each picking event, following the first ripening detection. This information was used in the calculation of the total yield per plant (YLD) for each harvest. Furthermore, the dynamics of the fruit load during harvest were evaluated through the percent distribution of yield (PDY) at each picking event (day of the year) and the cumulative percent yield (CPY) across picking events at every harvest.

### Physical fruit characteristics during ripening

Once fruit ripening (BBCH stage 88) was recorded for each effective plant, a sample of 10 fruits per evaluated plagiotropic branch was collected. For the collected fruits, the L*, a*, and b* parameters of the CIELAB color space were determined using a CR-410C colorimeter (Konica Minolta, Tokyo, Japan), and the color index (CI) was calculated using Equation 3 (Vélez-Sánchez et al., 2021). Additionally, epidermal firmness was measured at two points on the equatorial plane of the fruit using a PCE-PTR200 digital penetrometer (PCE-Iberica, Tobarra, Spain), and the fruit equatorial firmness (EF) was calculated as the mean. The fresh mass of the 10 fruits was recorded using an AR3130 electronic balance (Ohaus^®^, Mexico D.F., Mexico). The pericarp and bean (endosperm + embryo) of each fruit were subsequently separated and stored independently in paper bags, which were placed in an oven at 60 °C for 72 h until they reached a constant mass, at which point their dry mass was measured using an electronic balance. Furthermore, the fresh mass of fruit (M-F), pericarp dry mass (DM-P), bean dry mass (DM-G), and fruit dry mass (DM-F) were calculated for each unit.

(3) $$CI=(\frac{1000\;a^{*}}{L^{*}\;b^{*}})$$

where L* is the lightness, a* is the red/green coordinate, and b* is the yellow/blue coordinate in the CIELAB color space.

### Statistical analysis

Values obtained at the branch or plant level were summarized as medians per progeny and harvest for descriptive and inferential analyses. Initially, the descriptive analysis involved determining the underlying ripening clusters using the k-means method based on the standardized GDD values obtained per progeny for both the SH and MH. The number of clusters was determined using the “majority rule” across 30 methods (Niknafs, 2022) and by examining the cluster stability index with bootstrap (Hennig, 2023). A bivariate high-density region (HDR) plot was generated for the resulting clusters (Hyndman, 1996), along with dot plots to illustrate the dispersion of GDD per progeny at each harvest (Cleveland and McGill, 1984). This was performed to characterize the differences in the fruit ripening cycles of the progenies categorized by ripening clusters and to determine their distribution within the established clusters.

To identify the variables with the most important contributions to the factorial structure of each variable set—climatic (GDD, CTFR, PAR, R, RH, and T), source–sink relationships (GBL, SLA, LFR, F_Node, and YLD), and physical fruit characteristics (M-F, EF, DM-P, DM-G, DM-F, and CI)—a multiple factor analysis (MFA) was applied (Bécue-Bertaut and Pagès, 2008; Husson et al., 2025), with harvest (SH and MH) as the grouping factor. The variables were scaled to unit variance in the MFA. The contribution of each variable by grouping factor (e.g., GDD_SH and GDD_MH) to the two primary dimensions was evaluated relative to the expected mean value under the hypothesis of uniform contribution. Variables with contributions exceeding the mean value were considered relevant for both the SH and MH. MFA is a factorial analysis approach in which all variable groups are integrated by weighting each cluster in a balanced manner to obtain a global representation, and each group is described by a scalar product matrix defined over a set of individuals (Escofier and Pagès, 1994).

The inferential analysis of the variables selected via MFA for each variable set was defined on the basis of the strong association between variables within each set and the weak association between sets using Spearman’s correlation coefficient (γ) calculated per harvest as the criterion. The effects of ripening clusters, harvest time, and their interaction were evaluated for the climatic, source–sink relationship, and physical fruit characteristic variable sets using a modified semiparametric multivariate ANOVA-type method with repeated measures (Friedrich et al., 2019, 2023), with clusters as the between-subject factor and harvest as the within-subject factor. The p-value was evaluated using the parametric bootstrap (PBS) approximation (n = 10,000). This approach was used to determine whether the variability of relevant variables within each set depended on ripening clusters related to ripening cycle duration, harvest, or both. A second modified semiparametric multivariate ANOVA-type repeated-measures method was subsequently used for the climatic variable set, this time using the origin hybridization (Intra.H. or Inter.H.) as the between-subject factor and harvest (SH or MH) as the within-subject factor. This was conducted to determine whether the variability in fruit ripening cycles, as evaluated based on relevant climatic variables, was a function of hybridization (Intra.H. or Inter.H.), harvest, or both. Coupling the repeated-measures model with the semiparametric multivariate ANOVA-type method supports hypothesis testing across various factorial designs, with a sampling distribution that can be approximated by resampling techniques; this enables its use in cases with small sample sizes and does not require the assumption of normality or equality for the covariance matrix (Friedrich et al., 2019). A descriptive analysis using line plots differentiated on the basis of cluster and harvest was performed for variable sets for which the analysis of variance revealed significant differences for the interaction; moreover, tables were provided for factors with significant differences using mean and standard deviation alongside the median in parentheses.

The PDY variable was evaluated using a non-parametric method for longitudinal data following an F1-LD-F1 design structure, with clusters as the main factor (Noguchi et al., 2012), the picking event as the time factor, and their interaction also considered; progenies served as subjects observed at different time points. Statistical differences among clusters at each picking event were determined using 95% confidence intervals for the relative treatment effect (RTE) of the PDY. This was used to determine whether the variability in the fruit load over time was affected by the ripening clusters. Univariate rank-based non-parametric methods offer flexible and robust approaches for longitudinal data analysis when the assumptions of normality or equal covariance matrices are violated, distributions are skewed, outliers are present, or the sample size is small (Noguchi et al., 2012). Additionally, the CPY was described using line plots for ripening clusters across picking events for each harvest. In this section, the following packages were used: “factoextra” (Kassambara and Mundt, 2020), “NbClust” (Niknafs, 2022), “fpc” (Hennig, 2023), “ggplot2” (Wickham, 2016), “ggpubr” (Kassambara, 2025), “ggdensity” (Otto and Kahle, 2023), “FactoMineR” (Husson et al., 2025), “patchwork” (Pedersen, 2025), “rstatix” (Kassambara, 2021), “MANOVA.RM” (Friedrich et al., 2023), and “nparLD” (Noguchi et al., 2022). All packages were run using the R software version 4.4.1 (R Core Team, 2023).

## Results

**Figure 1:**
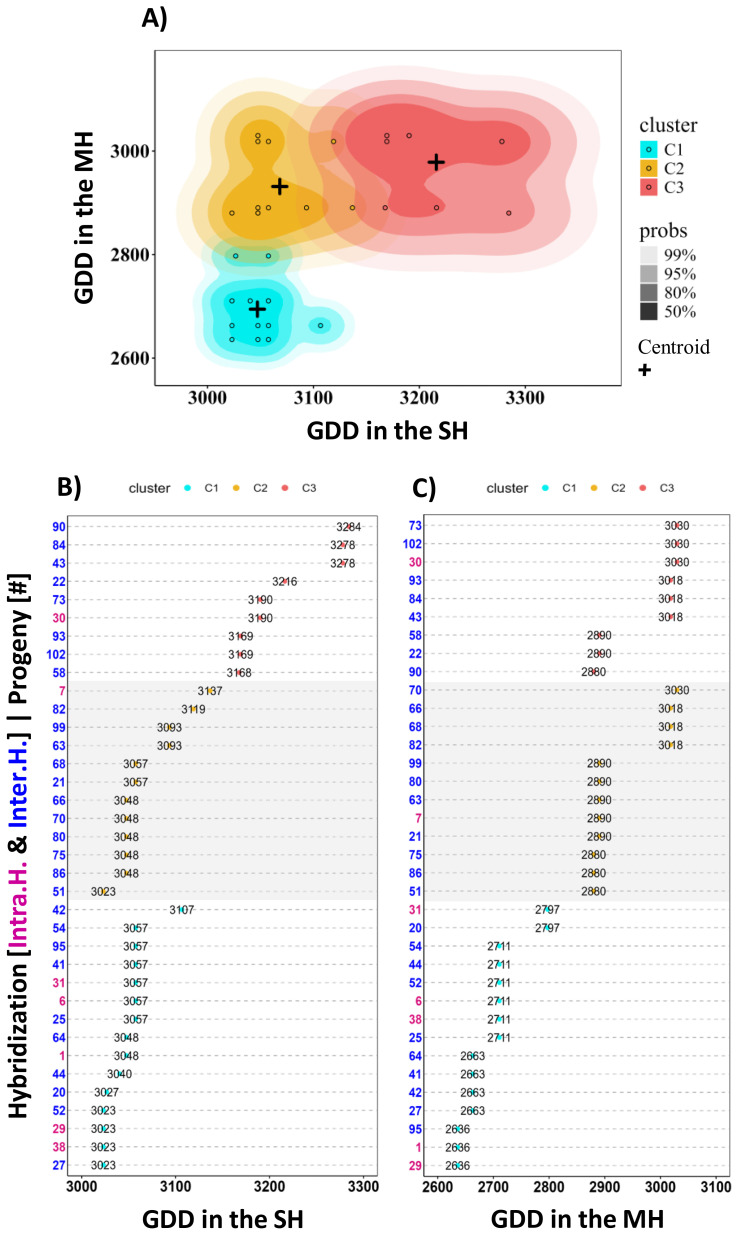
Bivariate distribution of high-density regions (HDRs) for growing degree days (GDD) between anthesis (BBCH stage 60) and fruit ripening (BBCH stage 88) recorded during the secondary harvest (SH) and the main harvest (MH) periods for ripening clusters [early ripening (C1), stable ripening (C2), and late ripening (C3)] **(A)**. Dot plots for GDD dispersion based on progeny number and hybridization [intraspecific *Coffea arabica* (Intra.H.) or interspecific *C. arabica* with *Coffea canephora* (Inter.H.)] and differentiated on the basis of ripening cluster for the SH **(B)** and the MH **(C)** periods. BBCH, Biologische Bundesanstalt, Bundessortenamt und CHemische Industrie.

**Figure 1A** illustrates the HDRs for the ripening clusters obtained via the k-means method using the GDD of the progenies from Intra.H. and Inter.H., calculated by harvest. The cluster (C1) represents early-ripening progenies, whereas the cluster (C3) represents late-ripening progenies (**Figure 1A**). These clusters differed significantly, as their HDRs did not overlap (99% probability) (**Figure 1A**). Early-ripening progenies (C1) had values close to 3,047 GDD for the SH and 2,695 GDD for the MH (centroid), whereas late-ripening progenies (C3) had values of approximately 3,216 GDD for the SH and 2,978 GDD for the MH (centroid) (**Figure 1A**). In both cases, the centroids for C1 and C3 were located within the HDRs with a 50% probability (**Figure 1A**). **Figures 1B, C** show the GDD for each progeny, determined on the basis of ripening cluster and hybridization type (Intra.H. and Inter.H.) for the SH and MH, respectively. C1 was associated with progenies exhibiting the lowest GDD values for both the SH (3,023–3,107 GDD) (**Figure 1B**) and the MH (2,636–2,797 GDD) (**Figure 1C**). This cluster exhibited the greatest difference in the GDD between harvests (ranging from 230 to 444 GDD) and accounted for 66.7% (10/15) of the Intra.H. progenies and 33.3% (5/15) of the Inter.H. progenies (**Figures 1B, C**). C1 accounted for 41.6% of the total evaluated progenies (**Figures 1B, C**). In contrast, C3 included progenies with the highest GDD for both the SH (3,168–3,284 GDD) (**Figure 1B**) and the MH (2,880–3,030 GDD) (**Figure 1C**), with differences ranging from 139 to 404 GDD between harvests. C3 was predominantly composed of Inter.H. progenies (8/9 = 88.8%) and constituted 25% of the total evaluated progenies (**Figures 1B, C**). These findings indicate that the early-ripening progenies in the SH period maintained this classification in the MH period, even though GDD accumulation was lower in the latter harvest stage; a similar observation was made for the late-ripening progenies. Furthermore, the majority of the evaluated progenies exhibited early ripening, whereas late-ripening progenies represented the minority and were primarily derived from Inter.H. crosses.

Furthermore, progenies in cluster (C2) exhibited behavior that could be defined as stable ripening, with centroid values near 3,068 GDD for the SH and 2,931 GDD for the MH, falling within the 50% probability HDR (**Figure 1A**). Nevertheless, two progenies categorized as C2 showed overlap with the 80% and 95% HDRs of C3 at the 80% probability level, a situation that could warrant reclassification in future studies (**Figure 1A**). The stability of GDD values for progenies in C2 resulted in performance similar to that of C1 during the SH period (3,023–3,137 GDD) (**Figure 1B**), whereas in the MH period (2,880–3,030 GDD) (**Figure 1C**), C2 progenies behaved similarly to those in C3. C2 encompassed 33.3% of the evaluated progenies, composed mainly of Inter.H. progenies (11/12 = 91.6%) (**Figures 1B, C**), while also displaying the lowest variation between harvests (18–247 GDD); consequently, these progenies were considered stable.

Generally, development from anthesis to fruit ripening required more GDD in the SH period (between 3,023 and 3,248 GDD) (**Figure 1B**) than in the MH period (between 2,636 and 3,030 GDD) (**Figure 1C**). All clusters exhibited multimodality, as shown by the HDRs with non-uniform concentrations (**Figure 1A**). Regardless of the ripening cycle duration across harvests, the performance of the progenies classified as C1 or C3 remained consistent (**Figure 1A**).

**Figure 2:**
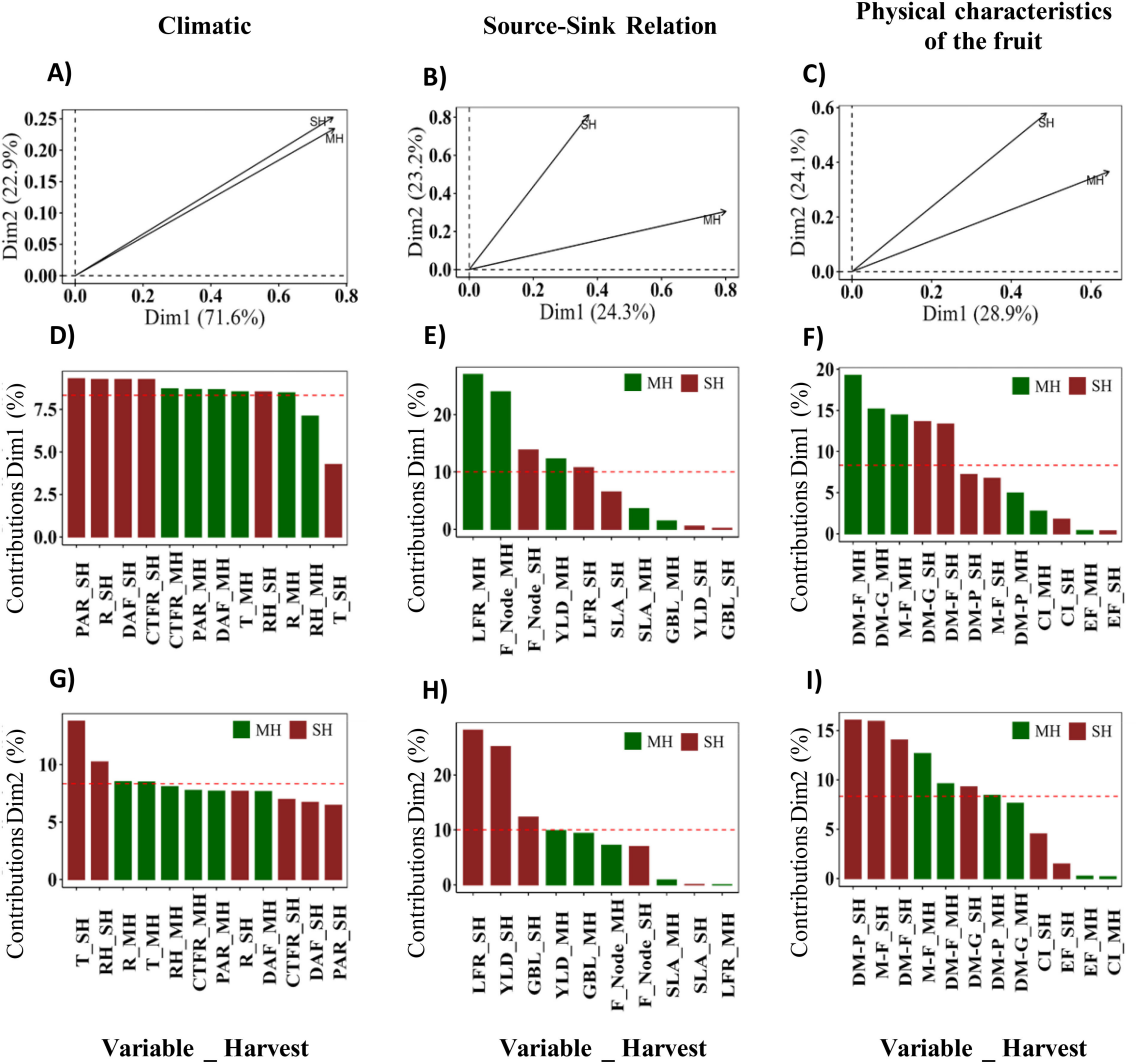
Vectors of the variable group **(A–C)**, contributions to the first dimension **(D–F)**, and contributions to the second dimension **(G–I)** of the multiple factor analysis (MFA) for the sets of climatic variables [growing degree days (GDD), chronological fruit ripening time (CTFR), photosynthetically active radiation (PAR), rainfall (R), relative humidity (RH), and average temperature (T)], source–sink relationship [absolute growth rate for branch length (GBL), specific leaf area (SLA), leaf area-to-fruit ratio (LFR), fruits per node (F_Node), and total yield per plant (YLD)], and physical fruit characteristics [fruit fresh mass (FM-F), fruit equatorial firmness (EF), pericarp dry mass (DM-P), bean dry mass (DM-G), fruit dry mass (DM-F), and color index (CI)] during the secondary (SH) and main (MH) harvests of coffee progenies. The red dashed line in the plots **(D–I)** corresponds to the expected mean.

**Figure 2** shows the MFA results for each variable set when harvest was used as the grouping factor. An analysis of the correlation structure for the climatic variable set revealed that the MH and SH vectors were closely aligned and situated near each other, indicating consistent variable behavior across harvests (**Figure 2A**). In contrast, the correlation structure for the source–sink relationship (**Figure 2B**) and physical fruit characteristic (**Figure 2C**) variable sets revealed that although the SH and MH vectors remained aligned, they were separated by a greater distance, indicating that variable behavior may differ between harvests. In the climatic variable set, Dimension 1 explained the greatest proportion of the variability (71.6%) (**Figure 2A**). Within this dimension, the variables GDD_SH, GDD_MH, CTFR_SH, CTFR_MH, PAR_SH, PAR_MH, R_SH, and R_MH were selected because they exceeded the expected average (red dashed line) (**Figure 2D**). In Dimension 2, although the variables RH_SH, RH_MH, T_SH, and T_MH presented values higher than the expected average, no variables were selected from this dimension, given its lower variance capture (22.9%) than Dimension 1 (**Figures 2A, G**). With respect to the source–sink relationship variable set, Dimension 1 captured 24.3% of the total variance (**Figure 2B**), where the variables LFR_SH, LFR_MH, F_Node_SH, and F_Node_MH were selected because their contributions exceeded the expected average (red dashed line) (**Figure 2E**). Dimension 2 captured 23.2% of the variance (**Figure 2B**), and within this dimension, the variables YLD_SH and YLD_MH were selected (**Figure 2H**). Finally, for the physical fruit characteristics variable set, DM-G_SH, DM-G_MH, DM-F_SH, and DM-F_MH were selected because of their significant contributions to Dimension 1, which accounted for 28.9% of the variance (**Figures 2C, F**). In Dimension 2, which accounted for 24.1% of the variance, FM-F_SH and FM-F_MH were selected because they contributed more than the expected average (**Figures 2C, I**).

**Figure 3:**
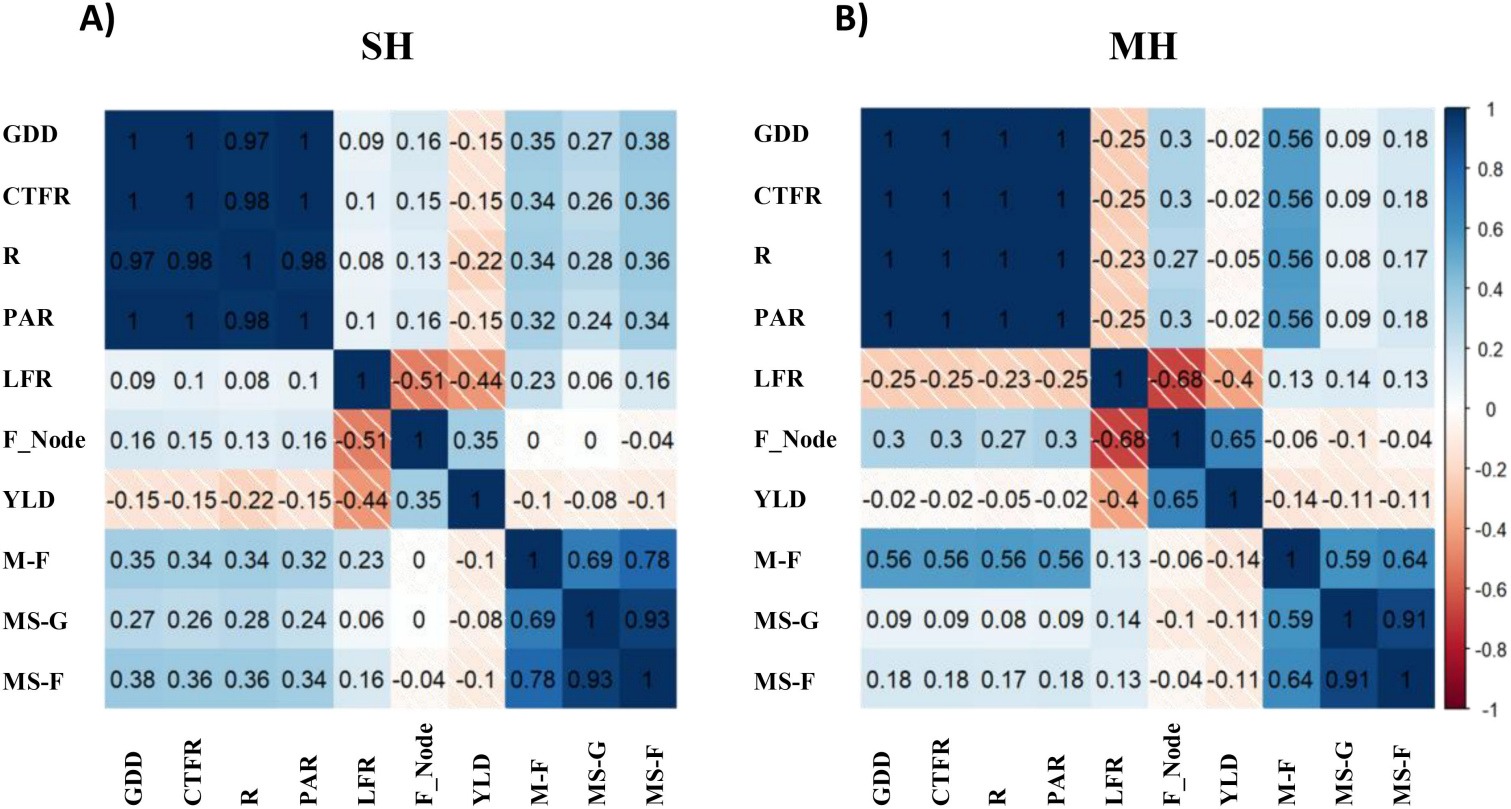
Heatmap of Spearman’s correlations among the variables: accumulated growing degree days (GDD), chronological fruit ripening time (CTFR), photosynthetically active radiation (PAR), rainfall (R), leaf area-to-fruit ratio (LFR), fruits per node (F_Node), total yield per plant (YLD), fruit fresh mass (FM-F), bean dry mass (DM-G), and fruit dry mass (DM-F) for the secondary harvest (SH) **(A)** and main harvest (MH) **(B)** of coffee progenies. The legend indicates the degree of correlation using the color scale in the heatmap.

A heatmap of the correlations between pairs of variables selected via the MFA revealed that, within each set, variables belonging to the climatic (GDD, CTFR, R, and PAR) and physical fruit characteristic (FM-F, DM-G, and DM-F) groups exhibited a strong association in both the SH (γ ≥ 0.69) (**Figure 3A**) and the MH (γ ≥ 0.59) periods (**Figure 3B**). In contrast, the source–sink relationship variables presented moderate-to-low associations in the SH (between γ = −0.51 and 0.35) (**Figure 3A**) and the MH (between γ = −0.68 and 0.65) periods (**Figure 3B**). Furthermore, inspection of the correlations of the selected variables between sets revealed that the associations were very low for the SH (between γ = −0.22 and 0.38) (**Figure 3A**) and moderate to low in the MH (between γ = −0.25 and 0.56) (**Figure 3B**). The strong association of relevant variables within each set in the MFA suggests that multivariate inferential analyses should be conducted independently for each set.

**Figure 4:**
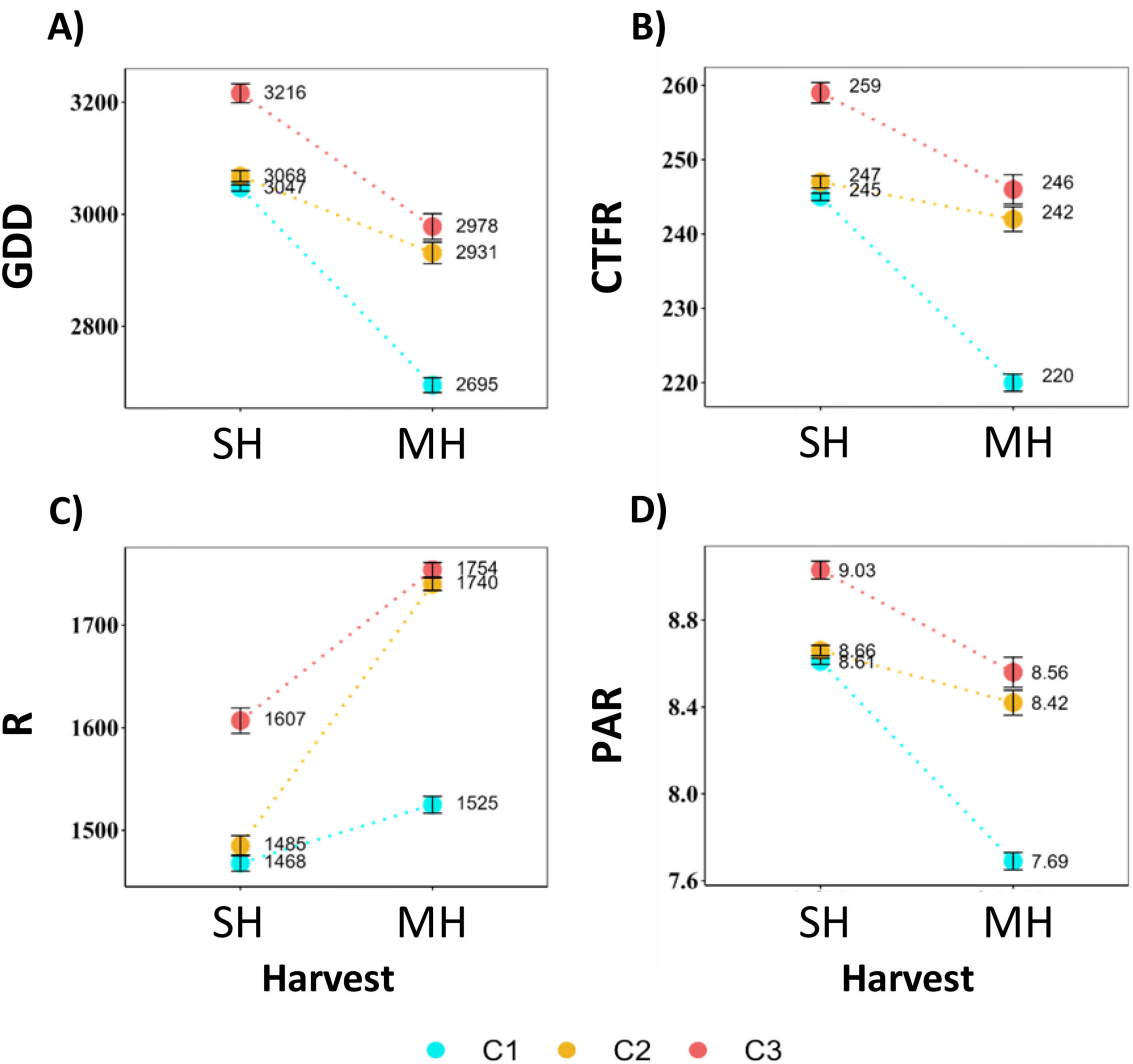
Means and their standard errors for growing degree days (GDD), chronological fruit ripening time (CTFR; DAF), rainfall (R; mm), and photosynthetically active radiation (PAR; 10^6^ mol m^−2^) for the early (C1), stable (C2), and late (C3) ripening clusters recorded during the secondary (SH) and main (MH) harvests for the coffee progenies.

**Table 2** shows the results of the modified semiparametric multivariate ANOVA-type analysis with repeated measures for each of the three variable sets (climatic, source–sink relationship, and physical fruit characteristics) using the ripening cluster as the between-subject factor and the harvest as the within-subject factor. The inferential analysis of the climatic variables (GDD, CTFR, R, and PAR) provided statistical evidence in favor of the alternative hypothesis, indicating statistical differences in the interaction (**Table 2**). These findings suggest that the differences in the GDD, CTFR, R, and PAR variables can be explained by both the established ripening clusters and harvest factors. Descriptive analysis revealed that for ripening clusters (C1, C2, and C3), the GDD (**Figure 4A**), CTFR (**Figure 4B**), and PAR (**Figure 4D**) decreased in the MH period compared with those in the SH period, whereas the opposite was observed for the R variable (**Figure 4C**). C3 had a higher GDD requirement to complete the ripening cycle, exceeding that of C1 by 5.2% during the SH and 9.5% during the MH (**Figure 4A**). Similar differences were observed between C3 and C1 for the CTFR (5.3% in the SH and 10.5% in the MH) and PAR (4.6% in the SH and 10.1% in the MH) (**Figures 4B, D**), with a slight increase in the differences for R (8.6% in the SH and 13.1% in the MH) (**Figure 4C**). C2 proved to be similar to the other clusters depending on the harvest; hence, there were statistical differences in the interaction (**Table 2**). In the SH period, C2 was similar to C1 and lower than C3, whereas in the MH period, C2 was similar to C3 and higher than C1 for the GDD, CTFR, PAR, and R variables (**Figures 4A–D**). However, the most relevant aspect of C2’s behavior was the slight differences between the SH and MH for the GDD (4.5%), CTFR (2.1%), and PAR (2.8%) compared with the values observed for C1 (GDD, 11.6%; CTFR, 10.3%; PAR, 10.6%) and C3 (GDD, 7.4%; CTFR, 5.2%; PAR, 5.1%) (**Figures 4A, B, D**). Therefore, C2 was labeled as having stable ripening. The only exception to this behavior was observed in the R variable, for which C2 showed a notable difference of 14.6% between the MH and SH periods (**Figure 4C**).

**Table 2:** Modified semiparametric multivariate ANOVA-type repeated-measures analysis with p-value estimation using the parametric bootstrap (PBS) approximation for the climatic variable [growing degree days (GDD), chronological fruit ripening time (CTFR), photosynthetically active radiation (PAR), and rainfall (R)], source–sink relationship [leaf area-to-fruit ratio (LFR), fruits per node (F_Node), and total yield per plant (YLD)], and physical fruit characteristic [fruit fresh mass (FM-F), bean dry mass (DM-G), and fruit dry mass (DM-F)] sets selected via multiple factor analysis (MFA) for coffee progenies.

Factor	Test statistic	PBS p-value
Climatic variables (GDD, CTFR, R, and PAR)		
Cluster	941.11	<0.001
Harvest	1,023.5	<0.001
Cluster × Harvest	309.31	<0.001
Source–sink relation (LFR, F_Node, and YLD)		
Cluster	9.614	0.25
Harvest	350.82	<0.001
Cluster × Harvest	3.522	0.657
Physical characteristics of the fruit (M-F, DM-G, and DM-F)		
Cluster	12.225	0.169
Harvest	16.295	0.012
Cluster × Harvest	9.356	0.171

The ripening cluster was the between-subject factor, whereas the harvest was the within-subject factor.

However, the inferential analysis of the source–sink relationship (LFR, F_Node, and YLD) factors revealed no significant evidence of differences between ripening clusters or their interactions. However, significant differences were identified for the harvest factors (**Table 2**). Similarly, for the physical fruit characteristics (M-F, DM-G, and DM-F), no significant differences were detected between the ripening clusters or their interactions. However, the null hypothesis associated with the harvest factor was rejected, although the differences were not extremely pronounced between harvests (p = 0.012) (**Table 2**). This finding indicates that variations in both the source–sink relationship and the physical fruit characteristic variable sets can be attributed solely to the harvest (SH vs. MH) rather than to the specific ripening cluster of the coffee progeny. The differences between the harvests are presented in **Table 3**. With respect to the variables associated with the source–sink relationship, LFR presented significantly greater values (71%) for HS than for the MH, whereas F_Node (63%) and YLD (84%) presented greater values for the MH (**Tables 2**, **3**). Among the variables associated with fruit characteristics, the SH was significantly greater for M-F (9%) and DM-F (2%), whereas DM-G (4%) was the greatest for the MH (**Table 3**).

**Table 3:** Descriptive statistics for climatic variables [growing degree days (GDD), chronological fruit ripening time (CTFR), photosynthetically active radiation (PAR), and rainfall (R)], source–sink relationship [leaf-to-fruit area ratio (LFR), fruits per node (F_Node), and total yield per plant (YLD)], and physical characteristics of the fruit [fresh fruit mass (M-F), bean dry mass (DM-G), and fruit dry mass (DM-F)] categorized on the basis of the secondary harvest (SH) and main harvest (MH) for coffee (*Coffea arabica*) progenies.

Variable	SH (n = 36)	MH (n = 36)
Climatic		
GDD	3,096.3 ± 78.4 (3,057.5)	2,844.5 ± 142.7 (2,880.2)
CTFR (DAF)	249.1 ± 6.5 (246)	233.5 ± 12.9 (237)
PAR (mol m^−2^)	8.73 10^6^ ± 1.93 10^5^ (8.63 10^6^)	8.15 10^6^ ± 4.37 10^5^ (8.27 10^6^)
R (mm)	1,508.5 ± 66.7 (1,490.1)	1,653.7 ± 113.8 (1,715.6)
Source–sink relation		
LFR (cm^2^ fruit^−1^)	14.22 ± 10.79 (10.27)	4.09 ± 3.00 (3.45)
F_Node (#)	5.3 ± 2.2 (4.9)	14.5 ± 4.3 (14.9)
YLD (g)	473 ± 333 (402)	2,900 ± 1,074 (2,989)
Physical characteristics of the fruit		
M-F (g)	1.91 ± 0.23 (1.89)	1.74 ± 0.17 (1.74)
DM-G (g)	0.388 ± 0.047 (0.394)	0.403 ± 0.045 (0.405)
DM-F (g)	0.571 ± 0.065 (0.576)	0.561 ± 0.052 (0.562)

The values correspond to the mean ± standard deviation (median).

DAF, days after flowering.

**Table 4** presents the results of the multivariate semiparametric modified ANOVA-type analysis for repeated measures of the climatic variables (GDD, CTFR, R, and PAR) using hybridization type (Intra-H. and Inter-H.) as the between-subject factor and harvest as the within-subject factor. This analysis revealed no significant differences in the interaction or between hybridization origins; however, differences were observed for the harvest factor (**Table 4**). Therefore, the differences observed in the GDD, CTFR, R, and PAR were not dependent on hybridization and were solely associated with harvest. **Table 3** presents the descriptive statistics for the selected climatic variables categorized by harvest. The SH exhibited significantly higher values for the CTFR (6%), GDD (8%), and PAR (7%) than the MH did, whereas the R variable was 9% greater for the MH (**Table 3**). These findings indicate that hybridization did not affect the duration of ripening cycles for the progenies. Furthermore, the MH harvest was characterized by shorter ripening cycles, attributed to lower accumulation of GDD, the CTFR, and PAR, which coincided with higher water availability than in the SH period, as reflected by an increase in R.

**Table 4:** Multivariate semiparametric modified ANOVA-type analysis for repeated measures with p-value estimation using the parametric bootstrap (PBS) approximation for the set of climatic variables [growing degree days (GDD), chronological fruit ripening time (CTFR), photosynthetically active radiation (PAR), and rainfall (R)] selected via multiple factor analysis (MFA) for coffee (*Coffea arabica*) progenies.

Factor	Test statistic	PBS p-value
Climatic variables (GDD, CTFR, R, and PAR)		
Hybridization	8.207	0.224
Harvest	129.01	<0.001
Hybridization × Harvest	3.292	0.104

The hybridization origin was the between-subject factor, and the harvest was the within-subject factor.

**Figure 5:**
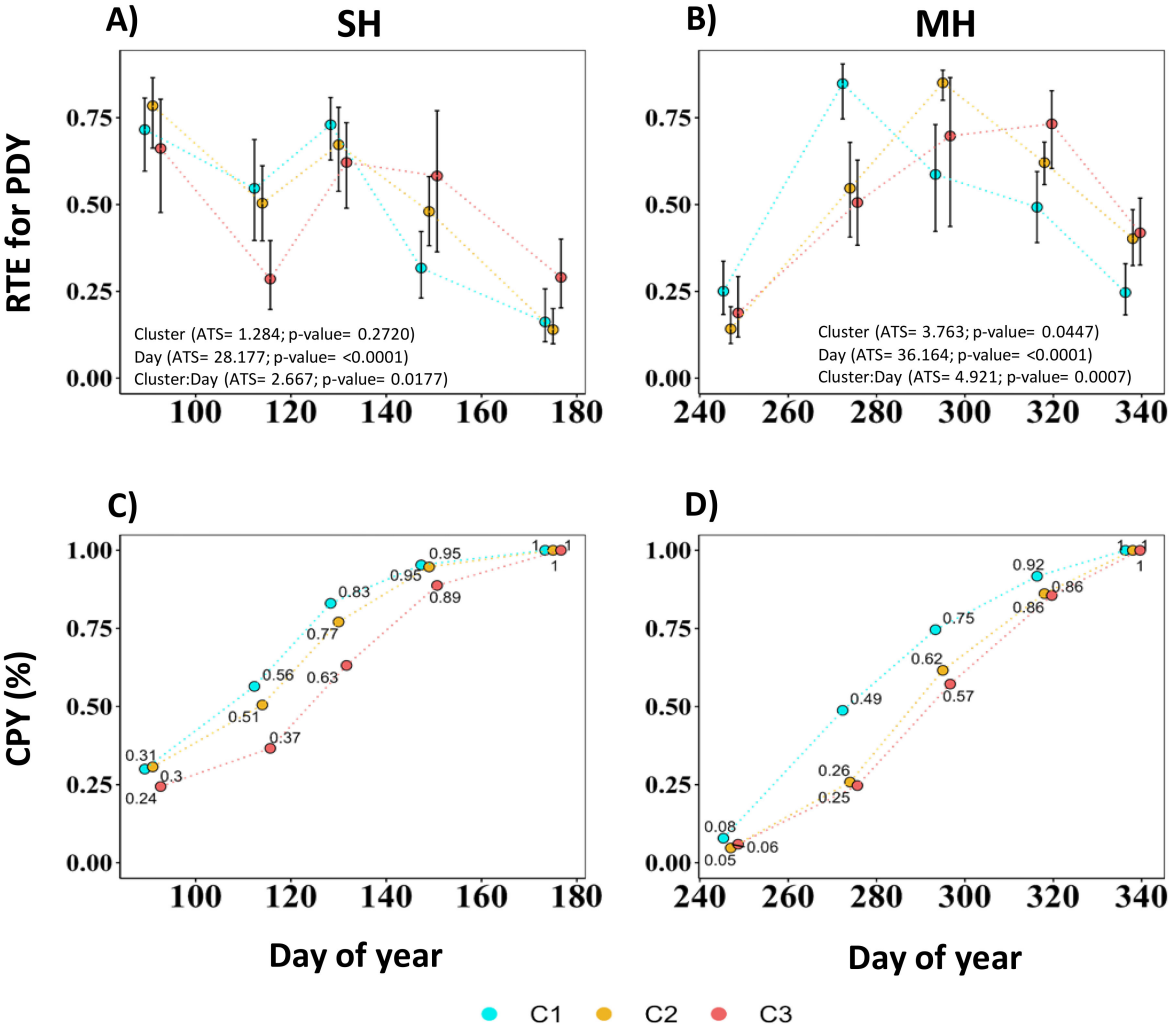
Relative treatment effect (RTE) and 95% confidence intervals calculated from the percentage distribution of yield (PDY) for the early-ripening (C1), stable-ripening (C2), and late-ripening (C3) clusters during the secondary harvest (SH) **(A)** and the main harvest (MH) **(B)** periods, including the corresponding non-parametric analysis with ripening clusters as the main effect, picking event day of the year as the time effect, and their interaction. Line graphs of the cumulative percentage yield (CPY) over time, categorized by ripening cluster for SH **(C)** and MH **(D)**.

Non-parametric analysis of longitudinal PDY data for each harvest (**Figures 5A,B**)—using ripening clusters as the main factor, picking events as the time factor, and their interaction—was used to evaluate fruit load dynamics. The analyses revealed significant differences in the interactions for both the SH (**Figure 5A**) and MH (**Figure 5B**). Consequently, the interaction between ripening clusters and each picking event was evaluated using 95% confidence intervals of the RTE for the PDY variable (**Figures 5A,B**). In the SH period, the RTE confidence intervals indicated that for the picking event on day 114, C1 and C2 had a higher load proportion than C3, whereas for the picking event on day 175, C3 was higher than C2 (**Figure 5A**). During the MH period, RTE confidence intervals showed that for the picking event on harvesting day 274, C1 was higher than C2 and C3; for the picking event on day 295, C2 was higher than C1; for the picking event on day 320, C3 was higher than C1; and finally, for the picking event on day 340, C3 and C2 exceeded the load proportion of C1 (**Figure 5B**). Generally, the changes in fruit load indicated that early-ripening progenies were characterized by a greater proportion of their fruit load during the initial picking events, which progressively decreased toward the final picking events. Conversely, late-ripening progenies exhibited a lower fruit load proportion during the initial picking events, with a gradual increase toward the final picking events. Cumulative fruit load dynamics were described using the CPY (**Figures 5C, D**). In the SH period, C2 exhibited a cumulative pattern similar to that of C1 (**Figure 5C**), whereas in the MH period, this behavior was similar to that of C3 (**Figure 5D**). This occurred despite the occurrence of multiple picking events throughout the season, which could have minimized the differential effect of the ripening cycle on the fruit load over time, but was shown to have no effect.

## Discussion

In the present study, coffee (*C. arabica*) progenies exhibited sufficient variability in terms of fruit ripening cycles on the basis of GDD to form three ripening clusters (early, stable, and late) (**Figures 1A–C**), which showed significant differences in multivariate analysis for climatic variables and their interactions (GDD, CTFR, PAR, and R) (**Table 2**). With respect to the CTFR, PAR, and R, a strong correlation with the GDD was observed because of the simultaneous recording of these variables (**Figures 3A,B**), as reflected in the consistency of the ripening behavior (**Figures 4A–D**). Water availability and solar radiation (W m^2^) are strongly associated with the GDD and CTFR (De Oliveira Aparecido et al., 2018; Da Silva Angelo et al., 2019). Progenies classified as C1 presented the lowest GDD accumulation at each harvest (**Figures 1A–C**, **4A**), whereas the highest GDD accumulation was observed for C3 progenies (**Figures 1A–C**, **4A**), as typically reported in previous studies (Petek et al., 2009; Nunes et al., 2010; Pezzopane et al., 2012; De Oliveira Aparecido et al., 2018; Da Silva Angelo et al., 2019). To ensure proportionality when comparing our GDD results with those in the literature, we used both 10.0 °C Tb and 10.5 °C Tb.

In this context, the GDD values for C1 during the MH period (2,636–2,797 GDD; Tb = 10.0 °C) (**Figure 1C**) were close to those previously reported for early-ripening *C. arabica* genotypes (Icatu Precoce IAC 3282, Mundo Novo IAC 464-12, IAPAR 59, Rume Sudam IAC 1139, and Costa Rica 95) (2,587–2,622 GDD; Tb = 10.0 °C) (Petek et al., 2009). Moreover, the GDD values for C3 during the MH period (2,880–3,030 GDD; Tb = 10.0 °C) (**Figure 1C**) were similar to those reported for genotypes (Obatã IAC 1669-20, Sarchímor IAPAR 88480-8, Tupi IAC 1669-33, Catuaí Vermelho IAC 99, Catucaí Vermelho 4-79, and Sarchímor E9702 III-1-9) with late-ripening characteristics (2,845–2,995 GDD; Tb = 10.0 °C) in Brazil (Petek et al., 2009). Furthermore, when a Tb of 10.5 °C was considered, the GDD values for C1 in the MH period (2,528–2,682 GDD; Tb = 10.5 °C) (**Supplementary Table 1**) were lower than those for the genotypes Catucaí 24/137 and Catucaí 785/15, reported as early-ripening varieties (2,827 and 2,899 GDD; Tb = 10.5 °C, respectively) (Da Silva Angelo et al., 2019). Nevertheless, the GDDs of these genotypes (Catucaí 24/137 and Catucaí 785/15) were consistent with the values obtained for C3 in the MH period (2,761–2,904; Tb = 10.5 °C) (**Supplementary Table 1**) and were slightly lower than those reported for the genotypes Arara and Palma III (2,921 and 2,940 GDD; Tb = 10.5 °C, respectively) (Da Silva Angelo et al., 2019) and Obatã IAC 1669-20 (3,090 GDD; Tb = 10.5 °C, 3,008 GDD; Tb = 10.2 °C) (Nunes et al., 2010; Pezzopane et al., 2012), which are classified as late ripening. This suggests that the designation of ripening type (e.g., early, late, or stable) depends on the genetic variability of the evaluated materials, which should be considered in future studies. The overall mean GDD in the MH period (2,844 GDD; Tb = 10.0 °C) (**Table 3**) of this study was comparable to that obtained for the *C. arabica* variety ‘Colombia’ (2,836 GDD; Tb = 10.0 °C) during the MH period in Colombia (Salazar et al., 1994) and slightly higher than that obtained in Brazil (2,781 GDD; Tb = 10.0 °C) (Petek et al., 2009). The mean GDD value in the MH period (2,727 GDD; Tb = 10.5 °C) (**Supplementary Table 1**), with a Tb of 10.5 °C, was lower than that calculated in Brazil (2,851 GDD; Tb = 10.5 °C) (Da Silva Angelo et al., 2019).

The aforementioned results indicate that progenies classified into C1 and C3 accumulated GDD during the MH within the ranges reported for *C. arabica* ripening cycles (Salazar et al., 1994; Petek et al., 2009; Pezzopane et al., 2012; Da Silva Angelo et al., 2019), highlighting the robust behavior of this trait even in equatorial regions. Notably, progenies derived from Inter-H. crosses did not exhibit the longer ripening cycles characteristic of *C. canephora* (Salazar et al., 2019), as may have been expected; instead, they maintained ranges similar to those of *C. arabica*. This could be attributed to the high degree of “Arabization” of the evaluated progenies, given their advanced filial generation (**Table 1**). This finding suggests that the fruit load in the MH period, which accounts for 86% of the total fruits harvested per year (**Table 3**), has similar implications for the duration of the ripening cycle as the single annual fruit load in Brazil does. In the central coffee-growing region of Colombia, the MH period (July–December), which involves multiple harvesting events, typically encompasses between 60% and 70% of the total fruits harvested annually (Arcila et al., 2007; Rendón, 2020). In contrast, Brazil has a single annual harvest between May and August, with 100% of fruit harvested during a single picking event (López et al., 2021; Ronchi and DaMatta, 2024).

Furthermore, there is no precedent in the literature for progenies classified as C2 (**Figures 1A–C**), mainly because this cluster was identified only through the evaluation of GDD during the SH period—a condition present in Colombia (Arcila et al., 2007; Rendón, 2020) but absent for fruiting in Brazil (López et al., 2021; Ronchi and DaMatta, 2024), where most studies of the *C. arabica* ripening cycle have been performed (Petek et al., 2009; Pezzopane et al., 2012; Da Silva Angelo et al., 2019). In this context, the GDD values for C2 (**Figure 1C**) were similar to those reported for the C3 progenies (**Figure 1C**) during the MH period; this may explain why this category had not been previously identified, in addition to exhibiting values within the reported range for *C. arabica* (Salazar et al., 1994; Petek et al., 2009; Pezzopane et al., 2012; Da Silva Angelo et al., 2019). To the best of our knowledge, the only record of stable ripening in coffee is for the *C. arabica* variety Caturra, whose GDD differed by 4.5% between the SH (2,560 GDD) and MH (2,445 GDD) (Jaramillo and Guzman, 1984). This difference was similar to that observed for the C2 progenies in the present study (**Figure 4A**). However, it is important to note that when the general range of the *C. arabica* ripening cycle is considered, the Caturra variety is classified as early ripening. This suggests the possible existence of other ripening cycle categories, such as “early stable” or “late stable”, which were not identified in this study. The presence of progenies in C2 resulted in significant differences in the interactions, as identified via multivariate analysis (**Table 2**; **Figures 4A–D**).

The progenies classified as C1 and C3 presented shorter and longer ripening cycles, respectively, than others at each harvest, although the cycle duration varied between harvests. In contrast, progenies classified as C2 exhibited less variation in cycle duration between harvests. This finding points to a differential response inherent to each progeny, with some being more sensitive to environmental conditions than others. The constitutively climacteric nature of coffee (*C. arabica*) fruits renders them more sensitive to endogenous signals than to environmental cues (Ságio et al., 2013; Da Silva Angelo et al., 2019), thereby maintaining a ripening response under diverse environmental conditions.

The ripening cycle duration exhibited by the progenies (**Figure 1**), defined through ripening clusters, showed no link with the set of source–sink relationship variables or physical fruit characteristics (**Table 2**). However, these should be interpreted with caution because the use of two replicates per progeny limited the test’s statistical power to detect subtle differences at a reduced sample size rather than true physiological independence, changing the results in larger samples, even though the statistical method has proven robust in small samples (Friedrich et al., 2019). A link between fruit ripening and the source–sink relationship has been reported for *C. arabica*; notably, plants with high yield (3,463 g) and a low LFR (8 cm^2^ fruit^−1^) without modifications to the initial fruit load ripened more slowly across harvesting events, regardless of whether they were in the shade or under full sun, compared with plants with one-quarter of the initial fruit load, a lower yield (1,698 g), and a higher LFR (20 cm^2^ fruit^−1^); this was based on a 50% difference in yield values and a 60% difference in LFR (Vaast et al., 2006). In comparison, in the present study, the difference in YLD between progenies in C1 and C3 was 26% for the SH and 9% for the MH, whereas for the LFR, the differences were 19% for the SH and 43% for the MH (**Supplementary Table 2**). In this regard, it should be considered that a link between the ripening cycle duration and the source–sink relationship may only occur when differences in YLD and the LFR exceed 50%, a condition that was not met among ripening clusters.

Furthermore, the variability caused by shifts in the fruit load between harvests indicates a probable link between ripening cycle duration and the source–sink relationship of plants. The multivariate analysis of the source–sink relationship (LFR, F_Node, and YLD) revealed a significant difference for the harvest factor but no such difference for the ripening factor (**Table 2**). This was due to significant increases in YLD (84%) and F_Node (63%) during the MH period compared with those in the SH period, which, in turn, resulted in a decrease in the LFR (71%) (**Table 3**). Although differences greater than 50% in yield and the LFR have been linked to ripening cycle duration, indicating that low fruit loads tend to accelerate fruit ripening (Vaast et al., 2006), these conditions contrast with the findings of the present study. The correlations among the LFR, F_Node, and YLD were moderate (**Figures 3A,B**). Similarly, the multivariate analysis of climatic variables (GDD, CTFR, PAR, and R) revealed significant differences for the harvest factor (**Table 4**), indicating increases in the GDD (8%), CTFR (6%), and PAR (7%) during the SH period compared with those during the MH period. In comparison, the MH was associated with an increase in R (9%) (**Table 3**). The differences in the source–sink relationship (LFR, F_Node, and YLD) stemming from the differential fruit load between harvests, along with differences in climatic variables between harvests, suggest a probable link between the fruit load per harvest and the extension of the ripening cycle, but in a different way than presented by Vaast et al. (2006).

From another perspective, Vaast et al. (2006) reported faster fruit ripening (by 1 month) for plants with full sun exposure, high fruit loads (1,880–2,700 g), and a low LFR (12–14 cm^2^ fruit^−1^) than for shaded plants with lower fruit loads (1,770–2,340 g) and a higher LFR (18 cm^2^ fruit^−1^). However, in that case, it was determined that the earlier ripening under full sun was due to a warmer microenvironment with higher irradiance rather than a direct effect of changes in the LFR. The moderate environmental radiation caused by shading increased the duration of the ripening cycle in *C. arabica* var. IPR99 plants (Morais et al., 2006). In this context, the increase in the fruit ripening cycle during the SH period could be linked to the reduction in microenvironmental temperature within the crop canopy, as reflected by the increased LFR due to the increase in leaf area (487 cm^2^) (**Supplementary Table 2**). This situation likely promotes increased evapotranspiration and radiation interception compared with those in the MH period, which presented a lower LFR and a smaller leaf area (338 cm^2^) (**Supplementary Table 2**). In *C. arabica* var. Castillo^®^, increasing the planting density resulted in an increase in average crop evapotranspiration during both the dry period (5.8 mm day^−1^) and the rainy period (5.2 mm day^−1^); in contrast, when the planting density was low, the crop evapotranspiration values were lower in both seasons (5.4 and 4.8 mm day^−1^, respectively) (Bermúdez-Flórez et al., 2018). In this way, differences in ripening cycles could be influenced by intra-canopy temperature variations, and indirectly for LFR, a hypothesis that warrants consideration in future studies, as microclimatic variables within the canopy were not directly measured in this study.

Consistent with the aforementioned data, the fruit load capacity of the plant during each harvest is reflected in the YLD variable, which, in turn, is linked to the observed variations in LFR and F_Node (**Table 3**). The difference in YLD between harvests (**Table 3**) was consistent with the shift in fruit load observed in the central coffee-growing region of Colombia (Arcila et al., 2007; Rendón, 2020). The change in LFR between harvests primarily resulted from the difference in fruit quantity (55.4%) rather than the difference in leaf area (30.5%) recorded along each branch (**Supplementary Table 2**). In this context, it has been reported that branch dry mass increases by 58.6% from anthesis to fruit ripening during the MH period, mainly because of fruit growth and defoliation resulting from high nutrient mobilization to the fruit (Sadeghian et al., 2025). LFR values in the SH period (**Table 3**) were similar to the LFR values (16.6 cm^2^ fruit^−1^) reported for low fruit loads (20%–50%) for *C. arabica* var. Cenicafé 1, whereas the LFR values in the MH period (**Table 3**) were close to the LFR (7.3 cm^2^ fruit^−1^) recorded under high fruit loads (60%–100%) (León-Burgos et al., 2024).

The decrease in the LFR observed during the MH period resulted in an increase in F_Node, which in turn reduced the physical space available for each fruit, consequently limiting individual growth and inducing changes in some physical characteristics, such as the reduction in M-F (9%) (**Table 3**). M-F has been reported to maintain a positive association with LFR in *C. arabica* var. Catuaí Vermelho IAC 44 (DaMatta et al., 2008), which is consistent with the results of the present study. The M-F values recorded in both the SH and MH periods (**Table 3**) were within the range documented for ripe *C. arabica* fruits in Colombia (1.51–2.29 g) (Buitrago-Osorio et al., 2022; Peñuela-Martínez et al., 2022; Osorio Pérez et al., 2023). The differences between the harvests observed in M-F were linked to minor changes in DM-F (2%) and DM-G (4%) (**Table 3**) and a more substantial change in DM-P (14%) (**Supplementary Table 2**). This suggests that the higher M-F and DM-F values recorded for the SH compared to those for the MH resulted from changes in DM-P. Slight differences in DM-F were observed between *C. arabica* var. Costa Rica 95 plants with a full fruit load and with a one-quarter load (Vaast et al., 2005). In the *C. arabica* variety Cenicafé 1, slight differences in bean size have been reported between plants with fruit loads of 50% and 90% (León-Burgos et al., 2024). The DM-F values found are within the range reported for *C. arabica* (0.41–0.64 g) (Salazar et al., 1994; Marín et al., 2003; Alberto et al., 2023; Osorio Pérez et al., 2023). The GBL decreased by 38% during the MH period because of the higher fruit load compared with that for the SH, which had a lower fruit load, whereas the SLA showed the opposite behavior, increasing by 13% between harvests and being higher in the MH period (**Supplementary Table 2**). These results align with those of previous studies that reported a positive association between fruit load and SLA and a negative association between fruit load and GBL (DaMatta et al., 2008; León-Burgos et al., 2024).

Statistical differences in the interaction found in the statistical analysis of the PDY for each harvest (**Figures 5A,B**), along with the descriptive differences in the CPY (**Figures 5C,D**), indicate that fruit load dynamics across picking events changed according to ripening clusters, replicating the behavior reflected by the GDD (**Figure 4A**). Thus, regardless of whether it was the SH or MH, the major picking events at the beginning of the harvest were concentrated in the progenies classified as C1 because of the lower GDD accumulation required to complete the ripening cycle (**Figures 5A,B**; **4A**). Conversely, the major picking events in C3 were concentrated toward the end of the harvest because of the higher GDD requirement to reach a ripe state (**Figures 5A,B**, **4A**). Furthermore, C2 included major picking events at the start of the harvest, similar to C1 during the SH period (**Figures 4A**, **5A,C**), but during the MH period, these events occurred toward the end of the harvest, similar to C3 (**Figures 4A**, **5B,D**), showing a behavior consistent with the observed GDD. The concentration of major picking events at the beginning of the harvest for early-ripening varieties and toward the end of the harvest for late-ripening varieties has been previously reported for *C. arabica* (Vaast et al., 2006; Ságio et al., 2013). This finding indicates that the ripening cycle directly influences fruit load dynamics throughout the harvest and that this effect is not diluted across multiple harvesting events during the season, a condition observed in Colombia, where multiple flowering events occur (Unigarro et al., 2025a). These results could broadly identify the ripening type (early or late) of progenies within large germplasm groups by evaluating fruit load dynamics across harvests in breeding programs. This is particularly relevant given that production records across harvesting events are routinely obtained for varietal selection, whereas records of fruit ripening cycles are scarce.

The absence of significant differences in the hybridization factor in the multivariate analysis of climatic variables (GDD, CTFR, PAR, and R) suggested that the duration of the fruit ripening cycle was independent of the hybridization type (Intra.H. or Inter.H.) (**Table 4**). However, the sample asymmetry between hybridization types may have influenced the lack of significant differences; therefore, these results were inconclusive and should be evaluated in future studies with symmetrical samples. Notably, longer ripening cycles were predominantly observed for progenies of Inter.H. origin (**Figures 1B,C**), possibly associated with the genetic influence of *C. canephora*, which typically has longer ripening cycles than *C. arabica*. The duration of the fruit ripening cycle of *C. arabica* (266 DAF) has been reported to be shorter than that documented for *C. canephora* (284 days) (Salazar et al., 2019), even considering the ranges recorded for early- and late-ripening progenies of *C. arabica* (213–254 DAF) (Da Silva Angelo et al., 2019) and *C. canephora* (216–300 DAF) (Partelli et al., 2014; Crasque et al., 2024, 2025).

## Conclusions

The findings of this study revealed that the behavior of progenies classified as early- and late-ripening remained consistent across harvests, despite the changes in the duration of the ripening cycle that all progenies experienced between harvest periods. Such changes in cycle length could be linked to the indirect effects of the source–sink relationship on the intra-canopy temperature; however, this relationship requires further investigation given the scope of this study. Furthermore, the behavior of the progenies classified as stable ripening was distinct, with only slight variations in the ripening cycle occurring between harvests. This finding indicates that progenies exhibit differential responses to environmental conditions, with some being more sensitive than others. Differences in ripening directly affect the dynamics of the fruit load over time, even under the specific conditions of Colombia, where multiple picking events could theoretically mitigate the effect of the ripening cycle. In this study, the duration of the ripening cycle was not affected by the physical characteristics of the fruit or the hybridization origin of the progenies. However, owing to the evaluated sample sizes, this first approximation should be interpreted with caution and verified in future studies. The results of this study offer an additional perspective for evaluating ripening cycles and provide fundamental information on their duration for different progenies. This is particularly relevant given the importance of ripening cycles in the context of harvest planning, beverage quality, and crop adaptability.

## Data Availability

The original contributions presented in the study are included in the article/**Supplementary Material**. Further inquiries can be directed to the corresponding author.
